# Affective Empathy in Behavioral Variant Frontotemporal Dementia: A Meta-Analysis

**DOI:** 10.3389/fneur.2018.00417

**Published:** 2018-06-12

**Authors:** Andrew R. Carr, Mario F. Mendez

**Affiliations:** ^1^V.A. Greater Los Angeles Healthcare System, Los Angeles, CA, United States; ^2^Department of Neurology, University of California, Los Angeles, Los Angeles, CA, United States; ^3^Departments of Psychiatry and Biobehavioral Sciences, University of California, Los Angeles, Los Angeles, CA, United States; ^4^David Geffen School of Medicine, University of California, Los Angeles, Los Angeles, CA, United States

**Keywords:** affective empathy, behavioral variant frontotemporal dementia, empathic concern, reactivity index, empathy

## Abstract

**Background:** Empathy deficits are a widely recognized symptom in the behavioral variant frontotemporal dementia (bvFTD), and although several reviews have examined cognitive empathy deficits, there are no meta-analytic studies on affective empathy deficits.

**Objective:** Identify salience of affective empathy in bvFTD.

**Method:** A thorough review of affective empathy found 139 possible studies, but only 10 studies included measures of affective empathy and met standardized criteria.

**Results:** BvFTD patients demonstrated a modest impairment compared to controls across all tasks (*d* = 0.98). Empathic concern as measured by the interpersonal reactivity index was particularly effected (*d* = 1.12).

**Conclusions:** This study provides evidence for an increased commitment to observing affective empathy in bvFTD and capturing its role in the disorder.

## Introduction

The behavioral variant of frontotemporal dementia (bvFTD) is neurodegenerative disorder that preys upon the social centers of the frontal and temporal lobes. Early in the progression, individuals with bvFTD demonstrate marked socioemotional behavioral disturbances including lack of insight, emotional blunting, and social disinhibition (1). One of the most problematic social changes is their loss of empathy as it deeply impacts their relationships (2). The loss of empathy is one of the five behavioral criteria in the International Consensus Criteria for the diagnosis of bvFTD (1). However, the concept of empathy itself is complicated and continues to be poorly defined in studies of bvFTD.

Empathy can be broadly defined as identifying with other's feeling states (3). More precisely it is an awareness of inhabiting an affect state corresponding to an affect state of another through observing or imagining that other's state (4, 5). This involves multiple affective experiences and includes emotional contagion or “affect sharing” in addition to affective perspective-taking, an extension of mentalizing (6). Thus, empathy is frequently broken down into affective and cognitive components, primarily: affect sharing and mentalizing (7). Given this characterization, we might have better insight into the type of empathy deficits that bvFTD patients demonstrate.

Several recent reviews and meta-analytic studies highlight the role of mentalizing, the basis of cognitive empathy, in bvFTD. Mentalizing or “Theory of Mind” (ToM) involves apprehending the thoughts (cognitive ToM) or feelings (affective ToM) of others. Lesion studies have indicated that ventromedial frontal lesions result in deficits in ToM and cognitive empathy (8). These deficits have been used to contrast bvFTD from Alzheimer's disease (AD). Bora et al. (9) reviewed 30 studies finding ToM deficits in bvFTD patients compared AD patients particularly in recognizing social faux pas. Clearly there is utility in examining cognitive empathy or mentalizing in this population. In a recent review by Henry et al. (10) of studies totalling 312 patients with bvFTD, they found significant difficulty with mentalizing tasks among these patients. More germane to this study, they found that emotion recognition played a salient role in studies despite not capturing affect sharing itself.

Whereas several robust reviews of mentalizing help to illuminate the cognitive impact on empathy, there are relatively few studies examining emotional empathy or affect sharing. Studies can evaluate emotional empathy by gauging aspects of affective empathy or the presence of visceral reactions to others affective states (7). For example, several studies of bvFTD patients indicate a greater level of emotional blunting and callous interactions with loved-ones (11–13).

Deficits in emotional or affective empathy most prominently arises from disturbances in the medial frontal cortex and the anterior insula (8); however, deficits and may also arise from disease affecting bilateral amygdala (14), precentral gyrus (15) orbitofrontal cortex (16), inferior parietal lobule, brainstem, and thalamus (17). Given that bvFTD has early and prominent medial frontal (including anterior cingulate) and anterior insula degeneration, these patients may have a pronounced impairment inn affective empathy. Additionally, affective empathy involves functional connectivity among the ventral anterior insula, orbitofrontal cortex, amygdala, and perigenual anterior cingulate (18). The white matter tracts such as the right uncinate fasciculus lesions may also be problematic in bvFTD empathy (19). Measures of affective empathy frequently come in the form of self or caregiver inventories. A common measure used for empathy is the Interpersonal Reactivity Index (20, 21). The subscale *empathic concern* assesses “other-oriented” feelings e.g., one's affective reaction to another's emotions. Previous literature have identified lower levels of empathic concern in bvFTD patients when rated by their caregivers (22–24); however, this is typically denied on self-reports (22). In one study (25) indicated that a reduced capacity for empathic concern in bvFTD is associated with relate decreases in left orbitofrontal cortex, left inferior frontal gyrus, left insular cortex, and the bilateral mid-cingulate gyrus.

Other measures of affective empathy involve direct behavioral tasks or observations. For example, the Picture Viewing Paradigms (26) attempts to capture affect sharing by having participants view an object or scene then report their level of distress or emotionality in response to the task. In another example, Oliver et al. (27), observed that bvFTD patients demonstrated lower levels of shared emotional experience, diminished arousal and more positive valence when viewing negative social scenarios. Finally, tasks with psychophysiological measures have been limited. One notable example demonstrates that bvFTD patients tend to have lower blood pressure than controls when viewing a video of a man completing a disgusting act (28). Across these studies, patients with bvFTD exhibit marked deficits sharing affective states of various stimuli.

We sought to summarize and evaluate the existing studies on affective empathy in bvFTD. The literature on affect sharing and empathic concern in bvFTD is reviewed. This quantitative review provides important point estimates that may clarify the magnitude of affective empathy deficits in bvFTD. Additionally, it can lead to recommendations for further investigation.

## Methods

### Literature search

We conducted a systematic review of the literature by searching the following databases: PubMed, Psych INFO, Web of Science, and Google-Scholar. The search consisted of the following terms: “bvFTD,” “bvFTD,” “FTD,” “empathy,” “experiencing sharing,” “affective empathy,” “prosocial concern,” “empathic concern,” “empathic motivation,” “IRI.” The literature search began March 3, 2017 and concluded June 11, 2017.

### Inclusion criteria

We chose studies based upon the following criteria: (1) the use of an accepted international consensus criteria for bvFTD (1, 29); (2) the presence of a non-bvFTD comparison group; (3) the presence of statistics necessary for the calculation of effect size, and (4) the use of a measure of affective empathy with a primary focus on emotion sharing. Multifactor empathy measures that incorporated cognitive theory of mind or perspective taking were excluded.

**Figure 1:**
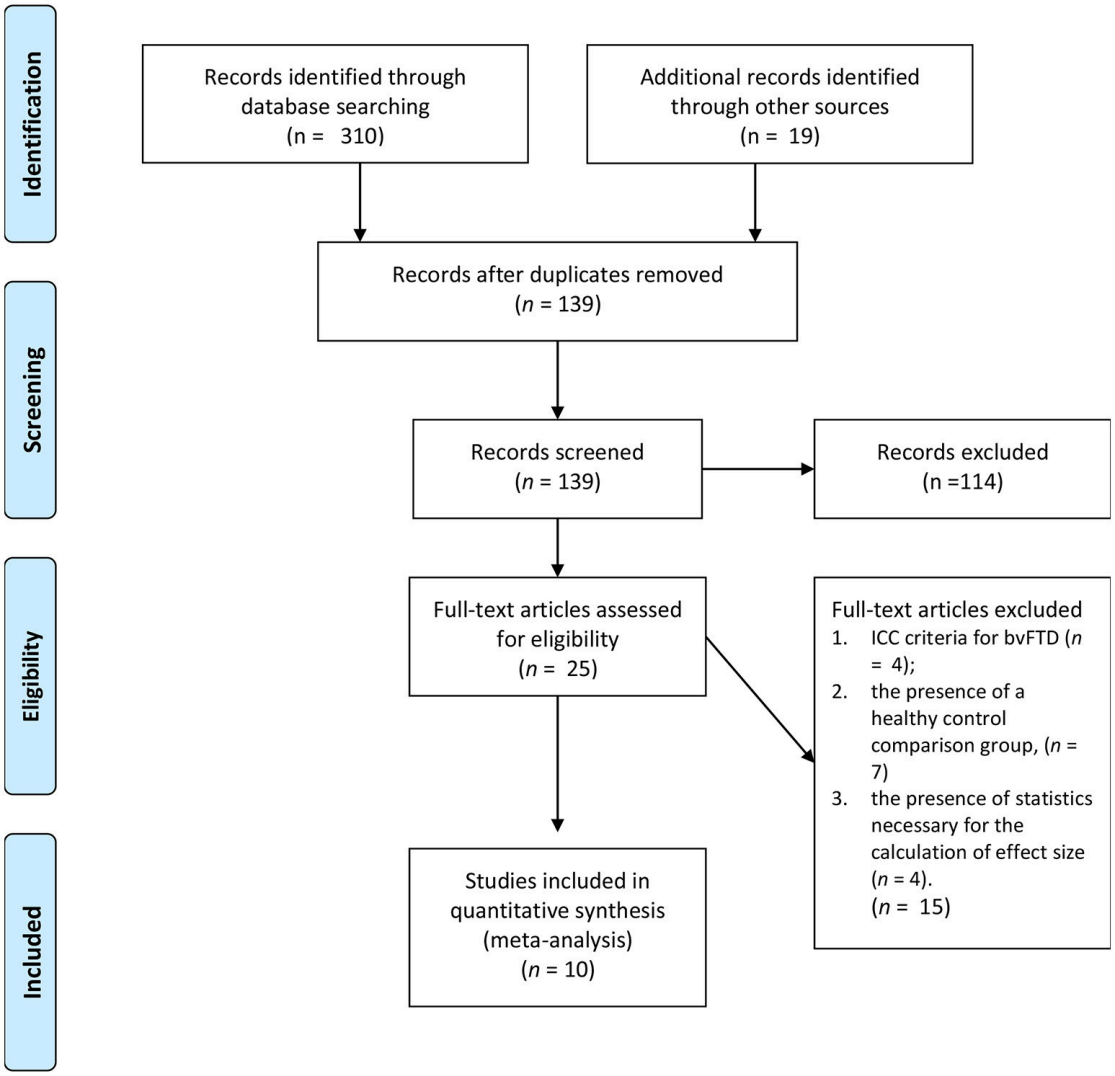
Literature selection process (30).

As see in Figure 1, the initial search yielded 139 studies across the databases. Only 25 studies met the first three criteria: (1) the use of an accepted international consensus criteria for bvFTD; (2) the presence of a non-bvFTD comparison group; and (3) the presence of statistics necessary for the calculation of effect size. Of those 25 only 10 included a measure of affective empathy with appropriate statistics present. Of those studies not selected empathy was characterized by emotional recognition or cognitive empathy task such as Reading the Mind in the Eyes Task (31–41). The characteristics of the included studies are included on Table 1 and the demographic information (Table 2).

**Table 1:** Characteristics of the publication.

**Authors**	**Publication status**	**Year**	**Journal**	**Variables**	
Tal Shany-Ur, Pardis Poorzand, Scott N. Grossman, Matthew E. Growdon, Jung Y. Jang, Robin S. Ketelle, Bruce L. Miller and Katherine P. Rankin	Published	2011	Cortex	CATS	
Diego Fernandez-Duque, Jodie A. Baird and Sandra E. Black	Published	2010	J Clinical and Experimental Neuropsychology	IRI-E	
Suzanne M. Shdo, Kamalini G. Ranasinghe, Kelly A. Gola, Clinton J. Mielke, Paul V. Sukhanov, Bruce L. Miller, and Katherine P. Rankin	In press	2017	Neuropsychologia	IRI-E	
Paul J. Eslinger, Peachie Moore, Chivon Anderson and Murray Grossman	Published	2011	J Neuropsychiatry Clin Neuroscience.	IRI-E	
Katherine P. Rankin, Maria Luisa Gorno-Tempini, Stephen C. Allison, Christine M. Stanley, Shenly Glenn, Michael W. Weiner, and Bruce L. Miller	Published	2006	Brain	IRI-E	
Marc Sollberger, Howard J. Rosen, Tal Shany-Ur, Jerin Ullah, Christine M. Stanley, Victor Laluz, Michael W. Weiner, Stephen M. Wilson, Bruce L. Miller and Katherine P. Rankin	Published	2014	Brain and Behavior	IRI-E	
Sharpley Hsieh, Muireann Irish, Naomi Daveson, John R. Hodges, and Olivier Piguet	Published	2013	J of Geriatric Psychiatry and Neurology	IRI-E	
Lindsay D. Oliver, Derek G.V. Mitchell, Isabel Dziobek, Julia MacKinley, Kristy Coleman, Katherine P. Rankin, and Elizabeth C. Finger	Published	2015	Neuropsychologia	Concern and Mirroring tasks
Sandra Baez, Facundo Manes, David Huepe, Teresa Torralva, Natalia Fiorentino, Fabian Richter, Daniela Huepe-Artigas, Jesica Ferrari, Patricia Montañes, Pablo Reyes, Diana Matallana, Nora S. Vigliecca, Jean Decety, and Agustin Ibanez	Published	2014	Frontiers in Aging Neuroscience	EPT-Concern rating
Paul J Eslinger, Peachie Moore, Vanessa Troiani, Shweta Antani, Katy Cross, Shaleigh Kwok, and Murray Grossman	Published	2017	J Neurol Neurosurg Psychiatry	Caregiver and Self ratings

*Comprehensive affect testing system (CATS), interpersonal reactivity index empathic concern scale (IRI-E) and empathy for pain concern rating (EPT-Concern rating)*.

**Table 2:** Demographics of studies.

**Authors**	**BvFTD**					**Controls**				
	***N***	**Age**	**Gender**	**Education**	**Severity**	***N***	**Age**	**Gender**	**Education**	**Severity**
(42)	39	61.6 (7.3)	26/13	15.7 (2.9)	26.6 (2.3)	77	68.2 (8.9)	32/45	17.6 (2.1)	29.4 (0.9)
(43)	9	62.3 (6.7)	7/2	16.2 (3.1)	27.0 (1.4)	10	65.4 (8.5)	6/4	16.0 (4.2)	29.0 (0.7)
(44)	58	60.8 (7.6)	39/19	16.4 (2.9)	23.8 (3.2)	44	68.7 (6.5)	15/29	17.2 (3.2)	29.3 (0.1)
(22)	12	<HC^*^	–	–	>HC^***^	12	>bvFTD^*^	–		<bvFTD^***^
(24)	30	59.5 (8.7)	23/7	16.0 (2.2)	1.2 (0.7)^t^	26	67.9 (5.3)	7/13	17.4 (2.7)	0^t^
(45)	28	62.4 (8.2)	21/7	16.4 (3.0)	25.9 (4.7)	19	71.3 (7.5)	7/12	17.6 (3.1)	29.6 (0.7)
(2)	18	63.4 (7.5)	13/5	11.3 (2.7)	6.0 (2.5)^t^	30	68.1 (5.6)	14/16	13.4 (2.7)	N/A^t^
(27)	24	64.7 (7.9)	12 /12	13.5 (3.1)	22.0 (5.1)	24	65.0 (8.5)	10/14	13.5 (3.3)	28.9(1.5)
(46)	37	66.0 (7.4)	15/22	13.68	25.92	30	55.0 (8.6)	15/15	14.6 (3.7)	28.31
(47)	26	69.16	–	14.78	29	17	75.07	–	15.14	29.33

*** *p < 0.01*,

* *p < 0.05*.

t *Studies measured severity using the Clinical Dementia Rating*.

### Outcomes

The primary objective of this review is to assess the impact of affective empathy in bvFTD. We include studies reporting the following outcomes. We examined affect matching evidenced by comprehensive affect testing system [CATS; (48)] and mirroring tasks (27). We also examined empathic concern ratings as evidenced by the interpersonal reactivity index empathic concern scale [IRI-E; (20)] and empathy for pain concern rating [EPT-Concern rating (49)]. We also explored outcomes for self and caregiver ratings as well as behavioral tasks.

### Statistical analysis

The authors combined the findings from the identified studies using the MetaEasy MS 1.04 Statistical package. Effect sizes were taken from pre-treatment measures in studies involving a repeated measure design.

Given the high likelihood of heterogeneity among the studies, the summary effect and 95% confidence intervals emerged from a random effects approach assuming both random and systematic error vary within the study's effect sizes using the DerSimonian-Laird (DL) approach (50). As such the Cochrane *Q* statistic tested heterogeneity and the *I*^2^ assessed the variation around the mean effect (51). Publication bias was assessed using Orwin's fail safe *N*, Egger's regression intercept.

## Results

**Figure 2:**
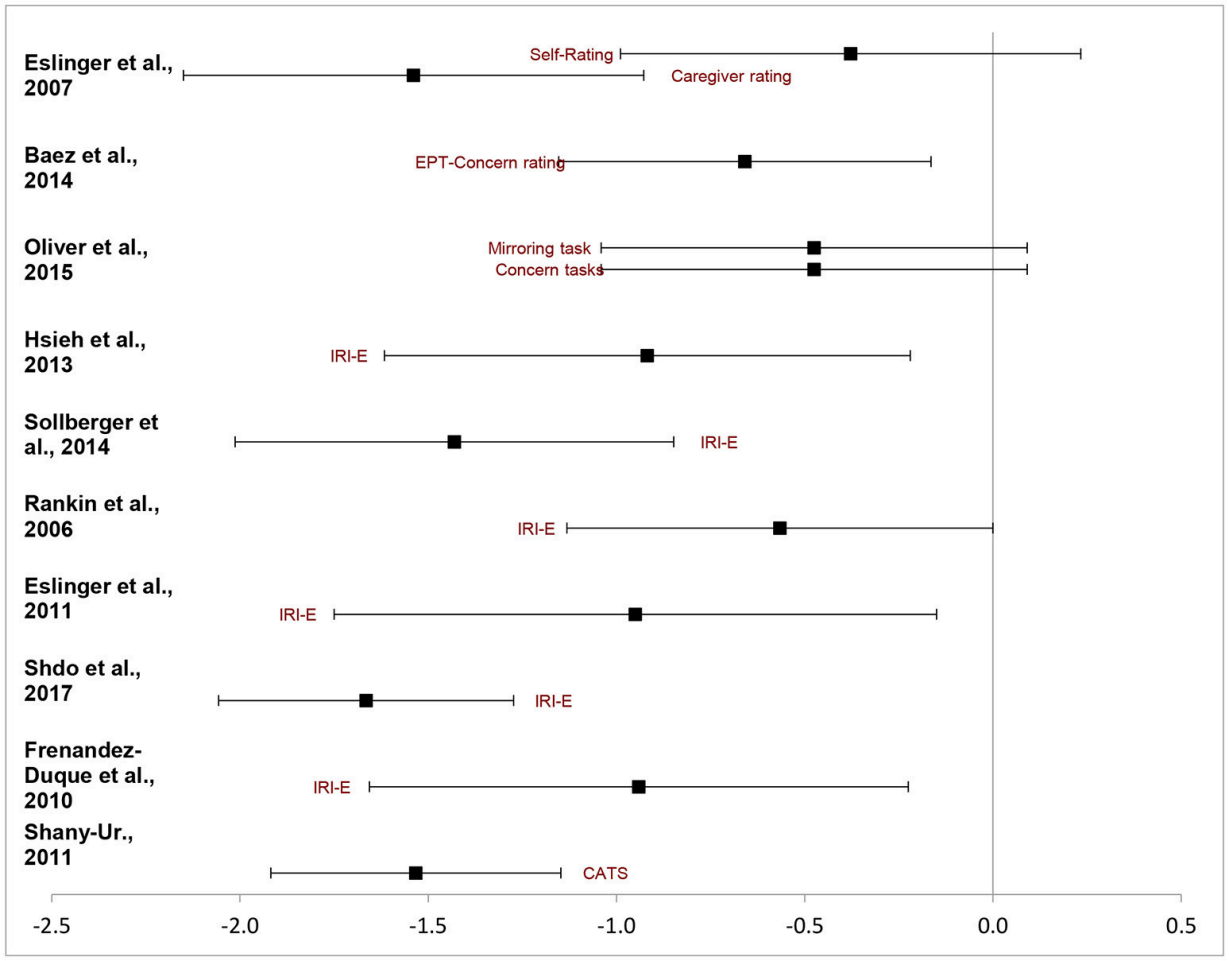
Random effect sizes. (2, 22, 24, 27, 30, 43–47).

Table 3 presents a study-by-study chart of the effect sizes on emotional empathy task in bvFTD relative to others. First an overall weighted mean effect size was calculated. A negative effect indicated the bvFTD group performed at a reduced ability compared to the reference group whereas a positive one indicated the reverse. The overall random effect DL model indicated a moderate effect size, *d* = 0.98 95%CI (−1.25, −0.71). Thus collapsed across all studies bvFTD patients are impaired in measures of emotional empathy compared to other non FTLD groups. Figure 2 depicts the ranges of the individual studies effect sizes.

**Table 3:** Effect sizes of studies.

**Authors**	**Effect**	**LCI95**	**UCI95**
(42)	−1.53	−1.92	−1.15
(43)	−0.94	−1.66	−0.22
(44)	−1.66	−2.06	−1.27
(22)	−0.95	−1.75	−0.15
(24)	−0.57	−1.13	0.00^NS^
(45)	−1.43	−2.01	−0.85
(2)	−0.92	−1.62	−0.22
(27) (Mirror)	−0.47	−1.04	0.09^NS^
(27) (Concern)	−0.47	−1.04	0.09^NS^
(46)	−0.66	−1.15	−0.16
(47) (Self)	−1.54	−2.15	−0.93
(47) (Carer)	−0.38	−0.99	0.23^NS^

*LCI95 indicates the lower limit of confidence interval and HCI95 indicates the higher limit of the confidence interval*.

NS *Not Significant*.

However, the analysis yielded significant heterogeneity amongst studies, *Q* = 30.72, *p*_q_ = 0.001. The *I*^2^ estimate indicated at 64 percent difference in random and systemic error between the studies. This could be attributable to the types of measures used and the variability within bvFTD behavioral presentations. Few clear subdivisions could emerge. When examining solely the effect size of the IRI-Empathic Concern scale a relatively similar pattern immerged. The five studies identified had an overall effect of *d*_*DL*_ = 1.12, 95%CI (−1.46, −0.08). However it too had a significant level of heterogeneity, *Q* = 12.33, *p*_q_ = 0.031. In each of these studies bvFTD patients had a more difficult time with emotional concern than their peers. On the other ratings listed, there was a strong effect for caregiver rated measures of empathy, *Z* = −1.54, 95%CI (−2.17 to −0.91) but no effect for self-rated empathy, *Z* = −0.38, 95%CI (−1.01, −0.91).

In terms of their performance on task-based measures of emotional empathy, the results were relatively mixed. On Oliver et al.'s (27) study in which participants view a video and provided a response to indicate their emotional concern and emotional there was a trend toward significance, 95%CI (−1.06, 0.11). However, the Shany-Ur affect matching test there was a strong deficit in the bvFTD group (*z* = −1.53, 95%CI (−1.92, −1.14).

## Discussion

The present meta-analytic study illuminates the magnitude of affective empathy deficits in bvFTD in the current published literature. This review examined the effects of 10 studies that depict impairments in affective empathy among patients with bvFTD. Given the magnitude of the effect size generated, it is likely that affective empathy plays a large role in the socioemotional alterations that characterize this disease. However, in addition to the paucity of studies, the overall heterogeneity issues across the samples indicate problems with measurement. Despite this, this meta-analysis indicates that the source of the impaired empathy in bvFTD extends beyond deficits in mentalizing to include significant primary deficits in affective empathy.

A lack of affective empathy is a central feature of bvFTD. This disorder is associated with neuropathology in areas of the brain that mediate affective empathy (52). These areas include medial frontal regions such as the ventromedial prefrontal cortex and anterior cingulate gyrus, the anterior insula, and associated areas such as the amygdale and the right anterior temporal lobe as well as corresponding neural networks according to Seeley et al. (53). It is not surprising that one of the main criteria and presentations of bvFTD is with impairments in expressions of empathy and sympathy toward other (54). These behaviors include a spectrum from simple lack of responsiveness to the concerns of loved ones to frank antisocial behaviors leading to trouble with society and the law (55). This meta-analysis supports this pathological and clinical profile of bvFTD.

Both rating scales and behavioral tasks find deficits in affective empathy. The tasks-based studies demonstrate clear problems with bvFTD patients' capacity to connect emotively with the world around them. These studies should be further replicated as they may produce insight to the individual behaviors within affective empathy, such as the lack of reciprocity in communication and the disconcerting prolongation of eye gaze (56). These compliment the robust inventories that speak to the day to day loss of affect connection which is a significant problem for family members of bvFTD (57).

The most frequent task used in the analysis was the IRI Empathic Concern scale. These studies indicated that caregivers generally feel a lack of warmth and connectedness to bvFTD patients. Although this caregiver assessment of empathic concern is only a proxy measure of affect sharing, it does indicate that bvFTD patients fail to convey affective empathy to those who know them. Given that an important evolutionary function of empathy is for prosocial connection (58), bvFTD patients fail to connect with the emotional experience of those they care about. Only the Rankin et al. (24) study failed to reach a significant effect size, which may be attributable to the use of older clinical criteria for bvFTD which has less specific socioemotional elements (29).

The task-based assessments yielded variable results. In the Shany-Ur study (42), the bvFTD patients had difficulty targeting the nonverbal aspects of affect sharing, and in the Oliver et al. study (27), the bvFTD patients did not show a significant effect. These studies differed in the required attention to nonverbal language processes and self-insight. Previous studies have shown that bvFTD patients have difficulty expressing their feeling states and lack the insight to know how well they are connecting to various social prompts (52, 59). They may instead report overlearned or social normed responses to various social situations (60). In other words, in scenarios with an easily detectable emotional prompt, like the Oliver et al study, they may respond typically, whereas when more subtly is involved in detecting emotionality from nonverbal aspects, as in the Shany-Ur study, they may disclose deficits in affect sharing.

This meta-analysis discloses several other findings from this research. One major takeaway is the paucity of studies using affective empathy as a core variable. Another finding is that most studies use a proxy measure such as a caregiver report. However, it is clear that direct task-based measures can be very informative in examining affective empathy in bvFTD. In particular, psychophysiological investigations of affective empathy can yield a more direct assessment of affective empathy among patients with bvFTD (12). Further connections to the basic sciences may help those studying early-onset dementias develop new paradigms for assessing socioemotional issues within this population.

As any study, this meta-analytic review is not without its limitations. Although many studies look at various aspects of empathy in bvFTD patients, there were surprisingly few that met all criteria. This is in large part due to the heterogeneity in research studies in this field. Often, exploring empathy within bvFTD patients is a secondary function of larger studies and uses crude measures to undertake such a task. More robust studies should be done to explore this as behavioral features are prominent in the diagnosis of this syndrome. Additionally, the use of a healthy control group excludes the typical comparisons of other dementia syndromes. Again, this was due to the low number of quality studies that involved multiple types of dementia patients. Future studies would do well to explore this more detail.

In conclusion, this review supports the presence of primary deficits in affective empathy among patients with bvFTD. Empathic concern, in particular, is a widely studied and broadly declined function in these patients. Future studies using task-based measures coupled with psychophysiological assessments and neuroimaging analysis would help further clarify this relationship and the brain-behavior mechanisms involved.

## Author contributions

AC participated in manuscript preparation, literature review, analysis, and formatting. MM participated in manuscript preparation, literature review, and analysis.

### Conflict of interest statement

The authors declare that the research was conducted in the absence of any commercial or financial relationships that could be construed as a potential conflict of interest.
